# The Interactive Factors Contributing to Fear of Death

**DOI:** 10.3389/fpsyg.2022.905594

**Published:** 2022-06-07

**Authors:** Mahdi Rezapour

**Affiliations:** Independent Researcher, Marlborough, MA, United States

**Keywords:** death fear, soul, religion, marital status, interaction terms, married life

## Abstract

Despite the importance of the topic of death, a limited comprehensive statistical analysis conducted highlighting the complex association between fear of death and various variables. Thus, this study is conducted to account for the possible complexity by considering all interaction terms after reducing the dimensionality of a dataset by means of recursive feature elimination, followed by the removal of the multi-collinear variables. The results highlighted, for instance, although being married, older and female offset the negative associations of fear of death, their impacts are multiplicative. Also, those who think cryonics is desirable are associated with higher fear of death. For instance, while belief in cryonics is positively associated with fear of death, its association varies depending on the trouble that individuals experience that someday they would not be alive and their marital status.

## Introduction

Unlike other species, the central perplexity of human beings is their awareness of the inevitability of death (Becker, 1997), where the inevitability of death itself fosters fear of death for human beings in both conscious and unconscious. Fear of death includes fear of own death, fear of the death process, and important others (Missler et al., 2012), while fear of the individual self-death is named as the core part of the death fear (Florian and Kravetz, 1983).

Studies are being conducted worldwide to investigate the fear of death to gain a better understanding of its associated factors and consequently reduce human suffering. These studies have been employed across a variety of contexts, such as religion, physical health, and quality of life. For instance, the relationship between religion and fear of death among older adults was investigated in a previous study (Fortuin et al., 2019). The results highlighted that religious people experience more death fear when compared to nonbelievers and highly religious people. In another study, the relationship between physical health and fear of death was investigated (Ding et al., 2020). The results revealed that physical health, meaning in life, and mental health are negatively associated with the fear of death.

Studies were also conducted to evaluate the costs of fear of death on the lives of human beings. For instance, death concerns and fear of death are associated with the trajectory and development of numerous psychiatric disorders (Iverach et al., 2014). In other studies, the role of death has been discussed in anxiety disorders, such as panic disorder (Fleet and Beitman, 1998) and obsessive-compulsive disorder (OCD) (Menzies and Dar-Nimrod, 2017).

In addition, it has been discussed that fear of death plays an important role in depression and psychosomatic disorders and psychopathology (Meyer, 1975; Feifel and Nagy, 1980). Many literature works discussed that most of the actions performed by human beings are to overcome that fear (Becker, 1997). Studying the fear component is particularly important, as fear is considered to be one of the most common responses of humans to death (Moore and Williamson, 2003). Based on the above-mentioned information, fear of death has many consequences, which are ultimately expected to reflect in the quality of life.

However, despite the efforts in the literature review, the majority of past studies failed to account for the multidimensional association between various variables and fear of death, which consequently hinders the ability to determine the real and valid associations across different predictors. In addition, a shortcoming of the majority of past studies is that they mainly focused on specific groups, rather than on the general population (Tsai et al., 2005). They also focused only on traditional methods, such as factor analysis, to solely describe variability among observed and correlated variables (Cicirelli, 1998), and thus they failed to highlight the most important features that should be incorporated in the analysis based on the prediction power.

Thus, to address the pre-existing limitation of past studies and to highlight reliable associations between important factors and fear of death, we first conducted a rigorous analysis to remove those variables that do not seem to be important for prediction across a myriad of factors, and then we excluded the multi-collinear variables. To summarize, the objectives of this study could be highlighted as follows:

1) Investigating the non-additive associations between death fear and other factors.
2) To have a better perspective about the underlying impacts of death fear, in addition to individuals' religious outlook, various behaviors and negative emotions were considered. For instance, religiosity or belief in God might not be necessarily important, but the underlying variables that seem to be related to religion should be considered instead. In other words, religiosity is not necessarily an indication of a belief in spirituality or the afterlife, so it is important to consider all those variables and let the algorithm pick important predictors.
3) Lastly, the concept of death and the human physical body are intricately tied (Moore and Williamson, 2003), so it is important to check how various emotions are tied with the physical condition of individuals. So, to address these concerns, specific types of questions regarding the physical aspects of the physical body were asked to the respondents, and the interaction between these variables was checked across all predictors.

In summary, in this study, we first exclude unimportant variables by employing recursive feature elimination (RFE). Then, to account for the possible multidimensionality of various variables, we account for all pairwise interaction terms. Also, we account for the structure of the dataset due to the inclusion of individuals' responses from different countries using the random-effect model.

## Methods

The responses were collected across various targeted countries, so the survey questions were translated by Qualtrics into Brazilian, Russian, Portuguese, Tagalog, and Hangul (Jong et al., 2019). Only those participants who passed the attention checks and did not leave out any section of the survey were incorporated into the study. No specific constraint was imposed on the populations, so the general population across countries was selected as the participants.

The participants were recruited in February 2015, and 2,058 participants attempted to start the survey. Out of the total number of participants, 37 were disqualified, as they were not from the specified countries. In addition, more than 680 individuals failed the attention test and hence were removed from the analysis. Informed consent was not provided by 408 participants, and so these were also removed from the analysis. The final dataset included a balanced dataset of 800 individuals across countries.

All the included questions were based on the main categories of the supernatural belief scale (SBS), death anxiety questionnaire (DAQ), and existential death anxiety scale (EDAS), which will be briefly discussed in the following section.

Questions related to supernatural beliefs, such as the afterlife or belief in the soul, are based on the supernatural belief scale (SBS), which examined the relationship between death anxiety and religious beliefs (Jong et al., 2019). In this study, the death anxiety was found to be associated with the religious identification of respondents, and when compared to non-religious individuals, religious participants experienced a lower magnitude of death anxiety. In this study, different supernatural beliefs (SBC) were considered, including the existence of God and subjective religiosity (Jong et al., 2019).

The DAQ is a 15-item scale that measures factors such as fear of suffering, loneliness, unknown, and personal extinction (Conte et al., 1982). The majority of our included variables were obtained from this scale, such as “It upsets me to think that someday I will no longer be in this world” or “The finality of death is frightening to me.” Similarly, the EDAS is a 12-item scale that measures death anxiety, targeting fear of non-existence.

Mortality preferences, and the concerns related to the physical body were evaluated by participants attitudes questions. For instance, response to questions like “How concerned are you with what happens to your body after you die?” Or the agreement to “specific mortuary arrangements such as cryonics preference” was considered. As discussed, fear of death includes the fear of own death and other important fears, such as fear of the process of death and fear of the unknown. Therefore, all these factors were considered, and their possible inclusion in the final model was left to the RFE algorithm.

**Figure 1:**
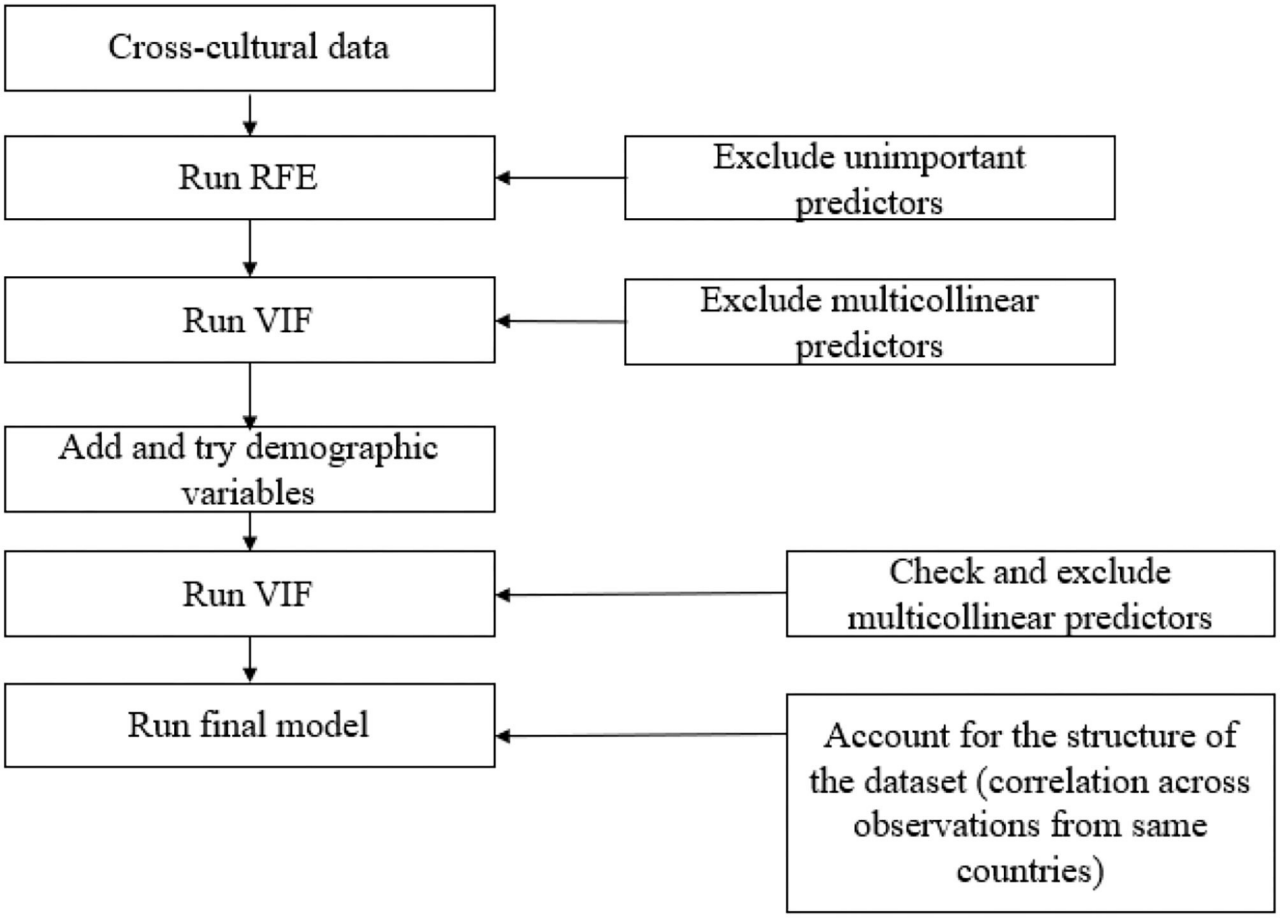
Methodological steps of the study.

The methodological steps are depicted in Figure 1. As can be seen from the figure, RFE was initially employed to incorporate only important predictors. It should be noted that RFE does not necessarily exclude the multi-collinear variables, so the variable inflation factor (VIF) was used to exclude the multi-collinear predictors after conducting the RFE and before running the final model. This step is particularly important, as the inclusion of multi-collinear variables is expected to result in biased and erroneous point estimates. Due to the importance of demographic characteristics, these variables were not incorporated in the initial analysis but were included after identifying the final predictors.

After highlighting the important demographic characteristics to be included in the model, VIF was employed again to make sure none of the variables are multi-collinear. Finally, the random-effect model or the hierarchical technique was employed to account for the structure of the dataset by taking into account the correlation across observations from the same countries. The survey questions included in the study were in accordance with the regulation of the Central University Research Ethics Committee (CUREC) of Oxford University (Jong et al., 2019).

Regarding the RFE technique, the number of variables was primarily set as 20, 30, 40, 50, 60, and 70. That is because after removing noisy variables, such as observation number, the maximum number of predictors was 70. The RFE algorithm considers the random forest technique and includes and excludes predictors to identify the most relevant variables suitable for inclusion in the model, and shows the highest prediction power. The RFE technique includes all variables in the dataset in the beginning and later computes importance scores and removes the weakest features. The model would be trained on the remaining features, and the importance score would be computed again and the weakest features would be removed. The process will continue until a desirable set of variables is reached.

For the final statistical method, a random-effect or hierarchical model was used. The technique is different from the standard linear model, as it accounts for the structure of the dataset by accounting for correlation across observations within the same category/country.

Again, RFE does not necessarily account for the significance or multicollinearity of variables. So, to include the best subset of predictors in the model, we first ran the RFE and then excluded those variables that were insignificant and multi-collinear. Few variables were excluded due to a lack of significance, such as “I am scared that death will extinguish me as a person.” The final model was based on the linear random-effect model.

## Results

The results of the descriptive statistics of variables are first presented, followed by the results of the statistical analysis.

### Variables

As discussed, the target was to obtain a response from 200 individuals from each country. Few variables were considered in the RFE, which were not found to be important in the statistical technique. These include predictors such as “Never feeling anything after death upsets me” or “Are you worried about not knowing what to expect after death.” All pairwise interaction terms were also considered and checked. Although many included predictors seem to be multi-collinear, all the final variables presented in Table 1 are not multi-collinear. For instance, consider the variables “The finality of death is frightening to me” and “Thinking about being dead fills me with dread.” Although these variables seem to be multi-collinear, no issue of multicollinearity was found (VIF < 0.5).

**Table 1:** Descriptive statistics of important predictors.

	**Type**	**Average**	**Variance**	**Min**	**Max**
***Response***					
The thought of my own death frightens me, (My_death_thought_frightens_me), < strongly disagree as −4, Strongly Agree as 4>	Likert	0.63	7.760	−4	4
***Demographic characteristics***					
Age, binary predictors, greater than mean (vs. others*), *Older (Freq = 341, Percentage = 43%)*	Binary	0.43	0.245	0	1
Gender, female (vs. male*) *Female (Freq = 422, Percentage = 53%)*	Binary	0.53	0.250	0	1
Marital status, married/committed (vs. others*) *Married/committed (Freq = 382, Percentage = 48%)*	Binary	0.56	0.246	0	1
***Supernatural***					
Every human being has a spirit or soul that is separate from the physical body (Human_has_spirit), < strongly disagree as −4, strongly agree as 4>	Likert	1.93	5.895	−4	4
There is some kind of life after death (Life_after_death), < strongly disagree as −4, Strongly Agree as 4>	Likert	1.68	6.274	−4	4
The loss of my consciousness in death scares me (Loss_of_consciousness_scares)	Likert	−0.04	8.223	−4	4
***Concerned with physical aspects of body***					
Mortuary practices: Cryonics (i.e., freezing your body), not at all Desirable 0– Extremely Desirable as 8	Likert	1.28	5.146	0	8
How concerned are you with what happens to your body after you die. (Your_body_after_death) < not at all concerned 0- extremely concerned as 6>	Likert	2.61	4.155	0	6
***Emotions***					
I am scared that death will be the end of me (Scared_death_is_the_end)	Likert	0.07	8.407	−4	4
It upsets me to think that someday I will no longer be in this world, (Upset_no_longer_in_the_world) < Strongly Disagree −4, Strongly Agree 4>	Likert	0.33	7.922	−4	4
Thinking about being dead fills me with dread (Death_fill_with_dread) < Strongly Disagree −4, Strongly Agree 4>	Likert	0.122	7.748	−4	4
My mortality troubles me (Mortality_trouble_me), < Strongly Disagree −4, Strongly Agree 4>	Likert	−0.146	7.970	−4	4
The finality of death is frightening to me (Death_finality_frightening) < Strongly Disagree −4, Strongly Agree 4>	Likert	0.20	8.388	−4	4
I am troubled by the fact that someday I will no longer be alive (Troubled_no_longer_alive)	Likert	0.23	8.084	−4	4
Do you worry about dying? (Death_worry ) 1–8 very much so	Likert	4.83	7.189	1	8
***Emotions in respond to others***					
Do you worry that those you care about may not remember you after your death? (Not_beimg_remembered), 1–8 very much so	Likert	3.70	7.810	1	8
Does it upset you to think that others may see you suffering when you die? (Others_see_you_suffering), 1–8 very much so	Likert	5.03	7.011	1	8

A few points should be elaborated on some of the included predictors. Age was categorized based on whether the participants were less than or equal to the average age of 34 years, as a reference, vs. others. Regarding marital status, we added the committed relationship to the married individuals. Our results are divided into a few main subsections. These categories include demographic, supernatural, concerned with physical aspects of the body, and emotional characteristics (Table 1).

Despite the importance of religion in the fear of death, this factor was not found to be important. Even after this variable was dropped by using RFE, we considered this predictor in the modeling process, but still found no significance. However, two factors, that is, believing in life after death and belief in the soul, were found to be important.

### Statistical Results

Our results in Table 2 are divided into a few main subsections similar to Table 1. While interpreting the results of interaction terms, all related main effects and interaction terms should be considered. Also, in the case of having variables considered in a few interaction terms, all those main effects and related interaction terms should be considered. For instance, consider the main effect of gender, where, for interpretation, we consider its main effect β_gender_ = −0.13, its related interaction terms of β_Gender × seperation of spirit and body_ = 0.08, and β_Gender × my mortaslity trouble me_= −0.07. Therefore, women experience less fear while interacting with other factors when compared to men. The results highlight that the association between fear of death and all predictors is multidimensional and that the impact of a single variable cannot contribute to the whole situation associated with fear of death (Table 2).

**Table 2:** Results of statistical analysis.

**Coef**.	**Est**.	**St. Error**	***p*-value**
(Intercept)	0.22	0.207	0.29
***Demographic characteristics***			
Age	−0.25	0.107	0.02
Gender	−0.13	0.130	0.33
Marital status	−0.15	0.119	0.22
***Supernatural***			
Human_has_spirit	−0.003	0.037	0.94
Life_after_death	−0.07	0.030	0.02
Loss_of_consciousness_scares	0.01	0.048	0.86
***Concerned with physical aspects of body***			
Cryonics (i.e., freezing your body)	−0.10	0.033	<0.005
***Emotions***			
Scared_death_is_the_end	−0.01	0.033	0.72
Upset_no_longer_in_the_world	0.13	0.033	<0.005
Death_fill_with_dread	0.18	0.032	<0.005
Mortality_trouble_me	0.26	0.059	<0.005
Death_finality_frightening	0.21	0.034	<0.005
Troubled_no_longer_alive	0.15	0.037	<0.005
Death_worry	0.11	0.027	<0.005
***Emotions in respond to others***			
Not_beimg_remembered	−0.06	0.021	0.009
Others_see_you_suffering	0.06	0.022	0.008
***Interaction terms***			
Age × Mortality_trouble_me	0.08	0.036	0.02
Gender × Human_has_spirit	0.08	0.041	0.06
Cryonics × Marital status	0.13	0.044	<0.005
Mortality_trouble_me × Loss_of_consciousness_scares	−0.02	0.008	0.05
Cryonics × Scared_death_is_the_end	0.03	0.010	<0.005
Upset_no_longer_in_the_world × Death_finality_frightening	0.03	0.009	<0.005
Death_finality_frightening × Death_fill_with_dread	−0.02	0.010	0.02
Cryonics × Troubled_no_longer_alive	−0.03	0.011	0.006
Mortality_trouble_me × Death_worry	−0.03	0.009	<0.005
Life_after_death × Human_has_spirit	0.02	0.008	0.03
Gender × Mortality_trouble_me	−0.07	0.036	0.06
Loss_of_consciousness_scares × Others_see_you_suffering	0.01	0.007	0.04

A better vision of the complex associations across various variables is shown in Figure 1. Due to complexity of the variables interrelationships, we had to move some variables around which might make the visualization process easier to follow.

**Figure 2:**
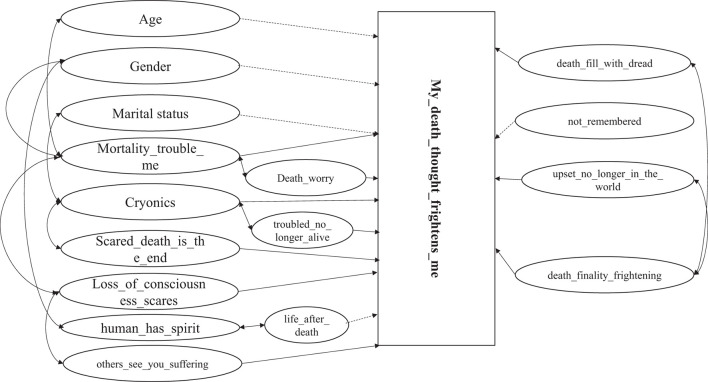
Complex interaction between factors and their association with fear of death.

In Figure 2, double arrows highlight the interaction terms, while the single-line arrows highlight the associations. As can be seen from Figure 2, there are important interaction terms across almost all predictors, except for the survey question, “Do you worry that those you care about may not remember you after your death?” Positive association is highlighted by solid lines, while the dashed line highlights the negative association, and these impacts were highlighted after considering all the related interaction terms. Also, all the impacts are related to the non-reference category in Figure 2. For instance, in Figure 1, the negative associations between female respondents and older age are all related to the non-reference category.

Due to the multiplicative impacts of variables, it can be seen that the association between items of cryonics and fear of death is sharper for those who are more afraid that death is the end of them. Also, when there is a positive association between “My mortality troubles me” and fear of death, the impact is lower for older respondents.

Herein, the interpretation of a few interaction terms will be outlined, and more elaboration will be presented in the discussion section. The interaction terms mean that the effect of one variable is different, which depends on the conditional distribution of the other variable. For instance, Age × Mortality_trouble_me interaction highlights that the estimated coefficients of ${\hat{\beta }}_{Age}=-0.2xs5$ and ${\hat{\beta }}_{\text{Mortality}\text{\_}\text{trouble}\text{\_}\text{me }}=0.26$ are so large when compared to their interaction term ${\hat{\beta }}_{\text{Age}\times \text{Mortality}\text{\_}\text{trouble}\text{\_}\text{me }}=0.08$, that those variable, while working in tandem, their impact work cover their interaction term. Consider the interaction of Gender × Human_has_spirit. Here, gender has the highest effect, ${\hat{\beta }}_{\text{Gender}}=-0.13$, when compared to ${\hat{\beta }}_{\text{Human}\text{\_}\text{has}\text{\_}\text{spir}}=-0.003$ , and their interaction term ${\hat{\beta }}_{\text{Gender}\times \text{Human}\text{\_}\text{has}\text{\_}\text{spir}}=0.08$. The results highlight that women experience less fear of death, and those respondents who believe in Human_has_spi experience higher fear. It should be noted that the scale of gender, being binary, and Human_has_spirit, being Likert, should be taken into consideration.

Also, it should be noted that if, for instance, the objective is the final change in the fear of death, all variables interacting with Human_has_spirit should be taken into consideration. For instance, similarly, Human_has_spirit is interacting with Life_after_death, where ${\hat{\beta }}_{\text{Human}\text{\_}\text{has}\text{\_}\text{spirit}\times \text{Life}\text{\_}\text{after}\text{\_}\text{death}}=0.02$. So by increasing Human_has_spirit by 1 unit, the fear of death increases.

## Discussion

Death creates a mixture of emotions, where due to the possible confounding effects, the associations between these emotions are not necessarily additive. The purpose of the current study was to understand the complex nature of various factors and their association with fear of death. We found an interactive effect across all factors, except for a single variable related to “not being remembered by those who we care about”.

### Supernatural Beliefs

Based on the past studies, belief in God or having religions are important for modeling death anxiety, and the previous studies also placed an emphasis on these factors, highlighting a negative association with death anxiety (Harding et al., 2005). However, in this study, these particular variables were not found to be significant for modeling fear of death. Even after dropping these variables by using the RFE algorithm, the variables and their interaction terms were considered and checked, but were still not found to be important. This finding might indicate that these beliefs are not necessarily associated with the fear of death.

Two supernatural beliefs were found to be important with opposite signs. These include belief in the soul and belief in life after death, which will be discussed in the following section. Counterintuitively, our findings fail to support the negative association, but found a positive association between belief in the soul and fear of death.

The second supernatural belief is related to the belief in the afterlife. It should be reiterated that despite numerous past studies associated belief in the afterlife with religion (Ellis et al., 2013), no significant interaction term was found across these factors.

A negative association between belief in the afterlife and fear of death is expected, as these individuals might see death as the beginning of a new existence, and it is expected that belief would help them to cope with concerns about finitude. It should be reiterated that the interaction between the afterlife and the belief in soul was also considered and found to be important, working in tandem, where belief in the afterlife and the soul are negatively and positively associated with fear of death, respectively.

The variation across the impacts of belief in the afterlife and belief in the soul might be due to higher associated doubt with the latter variable. The positive association between doubt and increased death anxiety was acknowledged in the past study (Henrie and Patrick, 2014), and a positive association between death anxiety and greater doubts about one's faith has also been discussed in the literature (Henrie and Patrick, 2014).

Although there is variation across fear and anxiety about death and also there is no proof that belief in separation between soul and body is accompanied by doubt, we hypothesized that the counterintuitive impact is likely to be due to the doubt (Upenieks, 2021). However, we concur and acknowledge that the result is counterintuitive and needs more investigation to better understand the underlying impacts of this association.

We did not drop the variable belief in soul from the analysis to motivate future studies for more investigation. It should be noted that belief in the soul was found to interact with gender, in addition to belief in the afterlife. In addition, it is worth noting that the positive association between fear of death and belief in the soul was mitigated for female individuals.

Although questions regarding the frequency of religious behaviors, or how often individuals discuss their religious beliefs with others, were also included in the survey questions, they were dropped by the RFE process as they were ranked to be less important.

### Cryonics

The result is an indication that those individuals who favor the method of preserving the body from decay and corruption experience more fear of death. This might be due to the lack of providing their ego with psychological ways of coping with the reality of ultimate dissolution (Minsky, 2013). It was interesting to see that the association between fear of death and cryonics varies based on the variables of marital status, being scared that death is the end of individuals' lives and being troubled by the fact that those individuals would be no longer alive after death. Also, while being married is the only variable with a buffering effect, the associations between other factors and fear of death are steeper for those individuals who agree with cryonics.

### Others See You Suffering and Not Being Remembered After Death

These two variables are directly linked to the “significant other,” but with opposite signs. The positive association between fear of death and others see you suffering is expected to be in accordance with the belief of knowing others are suffering from your suffering or extreme care and compassion for others.

On the other hand, although seems counterintuitive, compared to the positive association with the previous variable, noting that others who you care about might forget about you is negatively associated with fear of death. We hypothesize that variations across the two factors are due to the higher gravity of others see you suffering, compared to not being remembered by others only.

As a human being is a social being, it is expected that those who are having a higher degree of worrying about not being remembered after death are having a stronger connection and bonds with other individuals, which reduces the fear of death. That negative association is expected to be due to confounding factors, such as having kids, or other bounds that cause that association but were not considered in the survey questions. It should be noted that this is the only factor that was found to be not interactive.

### Age

Although fear of death is a concern for the entire course of human beings' lives, we found it to be a greater concern for the younger groups. The impact might be due to the fact that older adults have more wisdom and also are more mature to deal with the concept of death. That is in line with the past study which reported that as mental age increases, fear of death is expected to reduce (Lester, 1967). However, this observation is against the previous study which highlighted that it is a greater concern among the older adults of the United States, as they are more likely to contemplate the eventuality of death (Burke, 1991).

In addition, we observed that the association between age and fear of death was not stable but varied based on the factor of being troubled by mortality. In other words, the higher degree of being troubled by death aggregates the fear of death for both age groups.

### Marital Status

Regarding marital status, it was found that married individuals experience less fear of death, which is against the previous study (Firth-Cozens and Field, 1991). However, it should be noted that despite ignoring the interaction term of this factor in the past study, we included these terms and found them to be important.

It is expected that married individuals are more optimistic about the issue of death and consequently have less fear of death. We found that the negative association between marital status and fear of death varies based on whether cryonics is desirable for individuals or not, and the association with fear of death is higher for those individuals who agree more with the subject of cryonics.

### Limitations

Although rigorous examination of predictors was employed to better understand the underlying association between different variables, there remain a number of methodological limitations which need acknowledgment.

The negative association between fear of death and worrying about not being remembered after death by those who we care about might be due to other unobserved factors that the survey failed to account for. Although the considered interaction terms unlock the real associations, there are still many possible unobserved factors that might be ignored at the time of designing the survey. The inclusion of these variables could expand our understanding of the underlying association between that factor and fear of death. For instance, various questions regarding the number of kids or close bonds with friends should be considered in future studies.

On the other hand, considering cryonics might be related to other factors that the survey did not record. It could be objected that this factor is interactive with religion or belief in God, but the interactions between these factors were not found to be important. So, future studies should take into account the logical implications of each effect and try to consider these questions in their own context. For instance, the factor of the subjective health of individuals or their special attachments to their physical bodies could be good indicators to be considered for checking the interaction terms, thus highlighting a real impact.

In general, an important limitation of this study is that the survey failed to record the mental and physical health of individuals at the time of data collection. This is particularly important as these factors are expected to work interactively while impacting the fear of death. For instance, individuals with a disease might be expected to experience more worry and anxiety (Missler et al., 2012). A link between the higher mental health of individuals and higher optimism toward death and consequently lower fear of death was also investigated in the past study (Niemiec et al., 2010). Based on the above-mentioned findings, it is expected that these factors play important roles, and hence future studies should consider them.

The survey took into account more than 70 questions. Although there is no evidence that the subjects were negatively affected by the high dimensionality of the survey, future efforts should take into consideration the duration of the survey, so the participants would not be dissuaded from answering questions.

## Conclusion

Although fear has been considered a defense behavior that is basic to survival (Mountcastle, 1974), death is the basic reality of life, and the fear of that event results from a lack of certainty regarding life after death or other unknown events which might occur after death.

Individuals should be guided to actively seek value in life and establish meaning so that they can mitigate the fear of death. That is particularly important, as individuals with a strong meaning in life are associated with a lower fear of death (Routledge and Juhl, 2010). Based on our results, having a committed relationship or being married acts as a protective cushion against the fear of death.

In addition, various intervention strategies were discussed in the literature, including mindfulness exercise (Sullivan et al., 2009), and psychotherapy by means of constructing purpose and meaning in life (Snyder and Forsyth, 1991). Death education could help to meet the need of promoting reflection on existential themes and exploration of concerns regarding afterlife beliefs (Fonseca and Testoni, 2012), and consequently gaining a better reflection of meaning in life. Also, a healthy marriage could be emphasized in future studies for having a lower fear of death.

In summary, factors such as attachment to the body in terms of cryonics or various negative emotions due to the fear of death, such as having dread, being upset, troubled, frightened, and scared, positively associate with the fear of death. On the other hand, belief in life after death, being married, older, and female offsets the negative effects.

However, as discussed, the impacts of most variables are not stable but vary based on the impact of other predictors. Despite the importance of fear of death in the lives of individuals, little is known regarding the complex relationships between factors and fear of death.

## Data Availability Statement

The original contributions presented in the study are included in the article/supplementary material, further inquiries can be directed to the corresponding author/s.

## Ethics Statement

Ethical review and approval was not required for the study on human participants in accordance with the local legislation and institutional requirements. Written informed consent from the patients/participants or patients/participants legal guardian/next of kin was required to participate in this study in accordance with the national legislation and the institutional requirements.

## Author Contributions

The author confirms being the sole contributor of this work and has approved it for publication.

## Conflict of Interest

The author declares that the research was conducted in the absence of any commercial or financial relationships that could be construed as a potential conflict of interest.

## Publisher's Note

All claims expressed in this article are solely those of the authors and do not necessarily represent those of their affiliated organizations, or those of the publisher, the editors and the reviewers. Any product that may be evaluated in this article, or claim that may be made by its manufacturer, is not guaranteed or endorsed by the publisher.
